# CyberKnife for Recurrent Malignant Gliomas: A Systematic Review and Meta-Analysis

**DOI:** 10.3389/fonc.2021.652646

**Published:** 2021-03-29

**Authors:** Lucio De Maria, Lodovico Terzi di Bergamo, Alfredo Conti, Kazuhiko Hayashi, Valentina Pinzi, Taro Murai, Rachelle Lanciano, Sigita Burneikiene, Michela Buglione di Monale, Stefano Maria Magrini, Marco Maria Fontanella

**Affiliations:** ^1^ Unit of Neurosurgery, University of Brescia and ASST Spedali Civili, Brescia, Italy; ^2^ Institute of Oncology Research, Bellinzona, Switzerland; ^3^ Unit of Neurosurgery, Alma Mater Studiorum University of Bologna and IRCCS Istituto delle Scienze Neurologiche, Bologna, Italy; ^4^ Unit of Radiation Oncology, Osaka University Graduate School of Medicine, Suita, Japan; ^5^ Unit of Neurosurgery, Fondazione IRCCS Istituto Neurologico Carlo Besta, Milan, Italy; ^6^ Unit of Radiology, Nagoya City University Graduate School of Medical Sciences, Nagoya, Japan; ^7^ Philadelphia CyberKnife/Crozer Health, Havertown, PA, United States; ^8^ Boulder Neurosurgical Associates, Boulder, CO, United States; ^9^ Unit of Radiation Oncology, University of Brescia and ASST Spedali Civili, Brescia, Italy

**Keywords:** CyberKnife, stereotactic radiosurgery, malignant gliomas, recurrence, HGG, high-grade gliomas, glioblastoma, anaplastic astrocytoma

## Abstract

**Background and Objective:**

Possible treatment strategies for recurrent malignant gliomas include surgery, chemotherapy, radiotherapy, and combined treatments. Among different reirradiation modalities, the CyberKnife System has shown promising results. We conducted a systematic review of the literature and a meta-analysis to establish the efficacy and safety of CyberKnife treatment for recurrent malignant gliomas.

**Methods:**

We searched PubMed, MEDLINE, and EMBASE from 2000 to 2021 for studies evaluating the safety and efficacy of CyberKnife treatment for recurrent WHO grade III and grade IV gliomas of the brain. Two independent reviewers selected studies and abstracted data. Missing information was requested from the authors via email correspondence. The primary outcomes were median Overall Survival, median Time To Progression, and median Progression-Free Survival. We performed subgroup analyses regarding WHO grade and chemotherapy. Besides, we analyzed the relationship between median Time To Recurrence and median Overall Survival from CyberKnife treatment. The secondary outcomes were complications, local response, and recurrence. Data were analyzed using random-effects meta-analysis.

**Results:**

Thirteen studies reporting on 398 patients were included. Median Overall Survival from initial diagnosis and CyberKnife treatment was 22.6 months and 8.6 months. Median Time To Progression and median Progression-Free Survival from CyberKnife treatment were 6.7 months and 7.1 months. Median Overall Survival from CyberKnife treatment was 8.4 months for WHO grade IV gliomas, compared to 11 months for WHO grade III gliomas. Median Overall Survival from CyberKnife treatment was 4.4 months for patients who underwent CyberKnife treatment alone, compared to 9.5 months for patients who underwent CyberKnife treatment plus chemotherapy. We did not observe a correlation between median Time To Recurrence and median Overall Survival from CyberKnife. Rates of acute neurological and acute non-neurological side effects were 3.6% and 13%. Rates of corticosteroid dependency and radiation necrosis were 18.8% and 4.3%.

**Conclusions:**

Reirradiation of recurrent malignant gliomas with the CyberKnife System provides encouraging survival rates. There is a better survival trend for WHO grade III gliomas and for patients who undergo combined treatment with CyberKnife plus chemotherapy. Rates of complications are low. Larger prospective studies are warranted to provide more accurate results.

## Introduction

The majority of malignant brain tumors are represented by gliomas (70%) (1). The standard management of newly diagnosed malignant gliomas (MGs) is maximal resection followed by radiotherapy (RT) with concomitant and adjuvant chemotherapy (CMT) (2). Although a solid treatment strategy has been established for MGs, recurrence still occurs in almost all patients within 2 years after initial treatment (3–5). Possible treatment strategies for recurrent malignant gliomas (rMGs) include second-line CMTs, surgery with or without adjuvant therapies, and RT (2, 6, 7). Reirradiation appears to be an efficacious and safe treatment modality, providing survival benefits with acceptable risk (8, 9). Among different reirradiation modalities, hypofractionated stereotactic radiotherapy (HFSRT) has shown promising results as it allows delivery of a large total dose, in a precise target volume and short treatment duration (10, 11). Nowadays, various HFSRT and stereotactic radiosurgery (SRS) machines are available and their usage has been gradually increasing. All systems have excellent accuracy with targeting areas close to 1 mm (12–14). Among those, the CyberKnife® (CK) is a frameless image-guided radiotherapy system mounting a 6-MV linear accelerator on a highly maneuverable robotic arm (15). The CK System is a non-invasive and pain-free treatment strategy that requires a customized thermoplastic face mask, reducing patient discomfort associated with other frame-based radiosurgical systems. Unlike other SRS techniques, the CK does not require general or local anesthesia still ensuring a comparable level of accuracy (12). Particularly, the CK was found to have clinically relevant accuracy of 0.7 +/- 0.3 mm, minimizing normal brain radiation exposure and allowing for high doses of radiation to targeted areas (12, 16). Given its recent development, few case series have been reported on CK for rMGs of the brain, and indications are still debated. We hereby conducted a systematic review of the literature and a meta-analysis to provide physicians awareness about the efficacy and safety of CK treatment for rMGs.

## Materials and Methods

### Literature Search

The systematic review was performed according to the Preferred Reporting Items for Systematic Reviews and Meta-Analysis (PRISMA) guidelines (17). A comprehensive literature search of the databases PubMed, Ovid MEDLINE, and Ovid EMBASE databases was designed and conducted by an experienced librarian with input from the authors. The keywords “glioblastoma”, “anaplastic astrocytoma”, “malignant glioma”, “high-grade glioma”, “HHG”, “recurrence”, “recurrent malignant glioma”, “brain”, “CyberKnife”, “CK”, “stereotactic radiosurgery”, “SRS”, and “stereotactic radiotherapy” were used in “AND” and “OR” combinations. The search was limited to articles published between 2000 and 2021.

The following inclusion criteria were used: 1) English language, 2) case series reporting greater than 5 patients 3) studies reporting exclusively histologically proven World Health Organization (WHO) grade IV gliomas or WHO grade III gliomas of the brain (18), 4) studies reporting recurrence, and 5) studies reporting retreatment with the CK System at recurrence. The exclusion criteria were: 1) case series reporting fewer than 10 patients and case reports, 2) brain lesions other than MGs, 3) lesions not located in the brain (e.g. gliomas of the spinal cord), 4) studies reporting only newly diagnosed MGs, 5) studies reporting on irradiation techniques other than the CK System, 6) studies not reporting survival data.

Two authors determined the inclusion and exclusion criteria for the studies in the literature search. In studies with overlapping patient populations written by the same author/institution, we only included the largest or most complete dataset. In cases where outcomes were separated by WHO grade or CMT at recurrence, we abstracted outcomes separately to perform our subgroup analyses. Missing baseline data and outcomes information was requested from the authors via email correspondence. The authors of six included studies replied and the information provided was integrated into the data abstraction process.

### Data Extraction

For each study, we abstracted the following baseline information: number of patients; median age at CK treatment; gender; median Karnofsky Performance Status (KPS) at CK treatment; WHO grade and histotype at recurrence. Regarding treatment at initial diagnosis we collected information about: the extent of resection (EOR), i.e. gross total resection (GTR, resection of more than 99% of the preoperative tumor volume), subtotal resection (STR, 95%–99% resection); partial resection (PR, < 95% resection), and biopsy (B) (19); the number of patients who underwent conventional radiation therapy (CRT); and the number of patients who underwent CMT. About the recurrence interval, we abstracted the Time To Recurrence (TTR, the time span between initial treatment and CK) (20, 21). As for treatment at recurrence, we gathered the following data: median planned target volume (PTV); the median number of fractions; total radiation dose in Gray (Gy); the number of patients who underwent CMT.

### Objectives

Our primary endpoints were median Overall Survival (OS), median Time To Progression (TTP), and median Progression-Free Survival (PFS). As for OS, we extracted data from initial diagnosis (i.e. time-length from the date of initial diagnosis to death from any cause) and from CK (i.e. time-length from the date of the start of CK treatment to death from any cause) (22). Concerning TTP and PFS, we abstracted data from CK. The former was defined as the time elapsed between the start of CK treatment to Beside Recurrence (BR, new lesion developed after 4 weeks beside or inside the prescribed marginal isodose line of previous CK treatment) or Progressive Disease (PD, more than 25% growth of Gd−enhanced area within 4 weeks after CK treatment) (21). The latter was defined as the time elapsed between the start of CK treatment to any disease recurrence or death from any cause (23). For our subgroup analysis, we were able to abstract median OS from initial diagnosis and from CK treatment for WHO grade IV gliomas versus WHO grade III gliomas separately and for CK plus CMT versus CK treatment alone separately. Besides, we analyzed the relationship between median TTR and median OS from CK treatment.

The secondary endpoints were Local Response (LR), New Lesion (NL), and complications. The LR was assessed with Gd-enhanced Magnetic Resonance Imaging (MRI) at 1 month after CK treatment and was classified into the following categories: Complete Response (CR, Gd−enhanced area disappears and no regrowth is recognized for at least 4 weeks after treatment), Partial Response (PR, Gd−enhanced area is reduced by more than 50% and maintains this state for at least 4 weeks after treatment), No Change (NC, less than 50% reduction or less than 25% growth of Gd−enhanced area, maintained for at least 4 weeks after treatment) and PD (24). The development of NLs following initially controlled disease (i.e. CR, PR, NC), was divided into BR and Remote Recurrence (RR, lesion located remotely from the prescribed marginal isodose line of previous CK treatment) (25). Regarding complications, we extracted the number of acute neurological and non-neurological side effects, corticosteroid dependency (the onset of neurological deficits and/or cephalalgia requiring daily doses of dexamethasone > 4 mg for more than 8 weeks), radiation necrosis, and other toxicities (26).

### Study Risk of Bias Assessment

We modified the Newcastle-Ottawa Quality Assessment Scale to assess the methodologic quality of the studies included in this meta-analysis (27). This tool is designed for use in comparative studies; however, our analyzed studies did not have control groups, therefore, we assessed the study risk of bias based on selected items from the scale, focusing on the following questions: 1) Did the study include all patients or consecutive patients versus a selected sample? 2) Was the study retrospective or prospective? 3) Was clinical follow-up satisfactory, thus allowing ascertainment of all outcomes? 4) Were outcomes clearly reported? 5) Were there clearly defined inclusion and exclusion criteria?

### Statistical Analysis

We estimated each cohort’s cumulative prevalence and 95% confidence interval for each outcome. Event rates were pooled across studies using a random-effects meta-analysis. Heterogeneity across studies was evaluated using the I^2^ statistic. An I^2^ value of >50% suggests substantial heterogeneity. Meta-regression was not used in this study. For some outcomes it was not possible to estimate the standard errors, therefore a standard error of 0 was used in the meta-analysis. Pearson’s correlation was used to correlate median TTR and median OS from CK treatment. Statistical analyses were performed using OpenMeta [Analyst] (http://www.cebm.brown.edu/openmeta/) and R statistical package v3.4.1 (http://www.r-project.org).

## Results

### Literature Review

**Figure 1:**
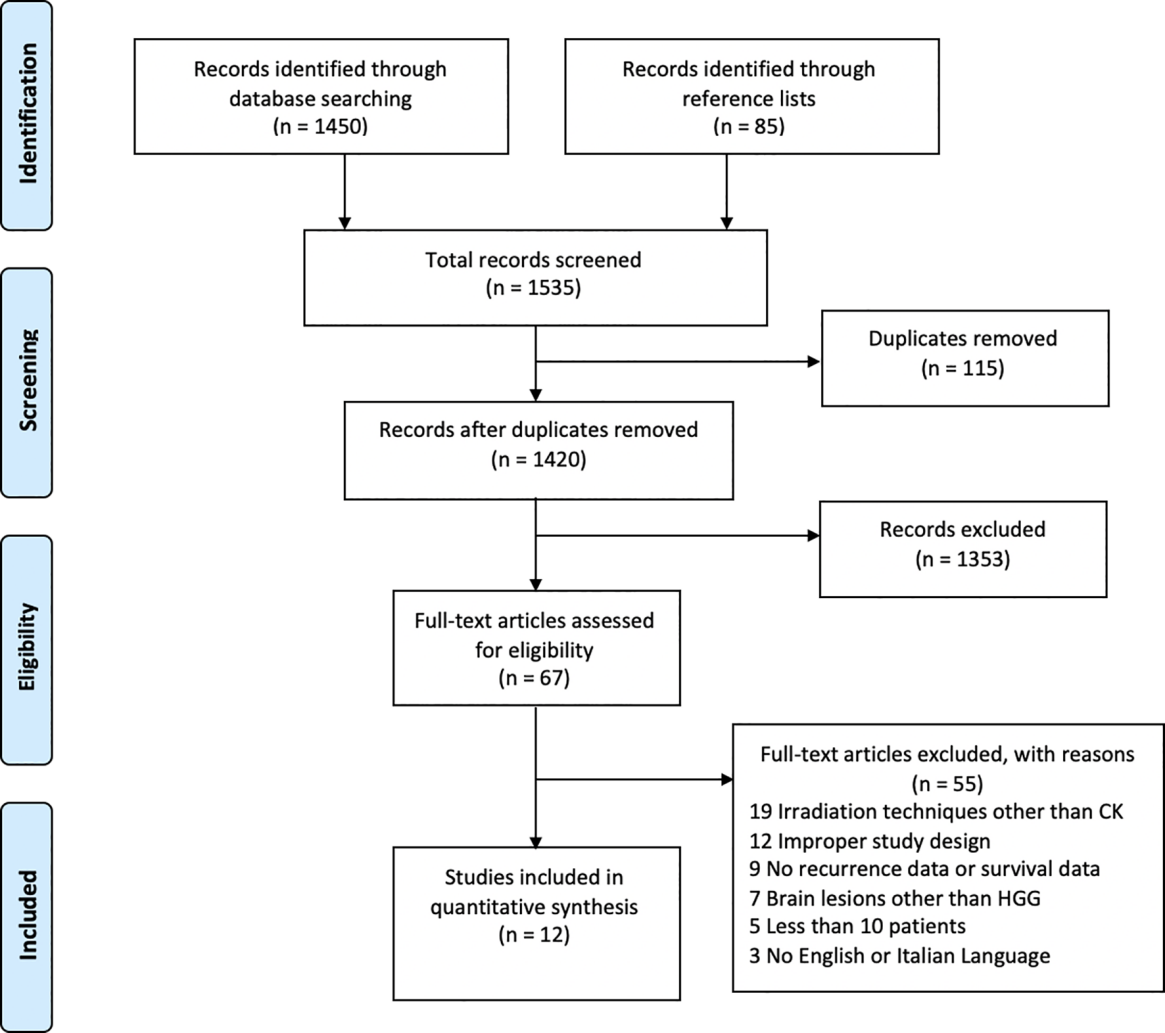
PRISMA flow-diagram depicting the literature search process.

A total of 1420 papers were identified after duplicates removal. After title and abstract analysis, 67 articles were identified for full-text analysis. Eligibility was ascertained for 12 articles (20, 21, 24–26, 28–34). The remaining 55 articles were excluded for the following reasons: 1) irradiation techniques other than the CK System (19 articles), 2) improper study design (12 articles), 3) studies reporting only on newly diagnosed MGs or not reporting survival data (9 articles) 4) studies reporting on brain lesions other than MGs (7 articles), 5) case series reporting fewer than 10 patients (5 articles), and 6) studies in other languages (3 articles). All studies included in the analysis had at least one or more outcome measures available for one or more of the patients’ groups analyzed. **Figure 1** shows the flow chart according to the PRISMA statement (17).

### Study and Patients Characteristics

Our meta-analysis included a total of 398 patients. The smallest study included 13 patients (32) and the largest included 128 patients (33). The median age at CK treatment was 54.5 years. There was a male predominance (1.6:1). The median KPS at CK treatment was 80. Six studies (50%) reported on WHO grade IV and III gliomas and other 6 studies (50%) reported on WHO grade IV gliomas. The histotype was available in 9 studies (75%): six studies (67%) reported on glioblastomas (GBMs) and 3 studies (33%) reported on GBMs and anaplastic astrocytomas (AAs).

At the time of initial diagnosis, most of the patients underwent STR (131, 33%), followed by GTR in 112 patients (28%), B in 30 patients (8%), and PR in 12 patients (3%). Post-operative CRT was undertaken in 343 patients (86%) and the median dose was 60 Gy. Post-operative CMT was undertaken in 275 patients (69%) and Temozolomide (TMZ) was the CMT regimen reported in most studies (193 patients, 48%). Patients were followed-up with a Gd-enhanced MRI performed every 1 to 3 months. The median TTR was 14 months (range 1-171).

At recurrence, the GTV was defined as the MRI Gd-enhanced area and the PTV was reconstructed adding 0 to 3 mm margin to the GTV. The median target volume (PTV) was 12.1 ml. The median number of fractions was 3 (range 1-6) and the median dose was 24.5 Gy (range 13.9-48.8). The prescribed marginal isodose ranged from 78% to 91%. Half of the patients (203, 51%) underwent CMT at recurrence. Although TMZ was the most reported CMT regimen (66 patients, 17%), other therapies were undertaken, particularly Bevacizumab (BEV) in 22 patients (5%) and Interferon in 16 patients (4%). Administration of CMT was concomitant and/or after CK treatment in 199 patients (98%) and before CK treatment in 4 patients (2%). The latter received BEV-based salvage therapy prior to CK treatment. A summary of the included studies is provided in **Table 1**.

**Table 1:** Summary of studies.

Study			Baseline Data						1st Treatment			Recurrence Interval	2nd Treatment			
No.	Author, Journal, Year	Prospective/ Retrospective	No. of Patients	Median Age; range	M:F	Median KPS; range	WHO Grade	Histotype	GTR/STR/PR/B (%)	CRT (%)	CMT (%)	Median TTR (months); range	CK			CMT (%)
													Median PTV (ml)	Median No. of Fractions	Median Dose (Gy)	
1	Adachi, Anticancer Research, 2019 (28)	R	29	53	19:10	75	IV-III	NA	NA	29 (100)	29 (100)	NA	4.1	1	25.5	29 (100)
2	Conti, Acta Neurochirurgica, 2012 (26)	P	23	58	13:10	80	IV	GBM	NA	23 (100)	23 (100)	NA	15.7	2	20	12 (52)
3	Ekici, J BUON, 2014 (29)	R	27	NA	NA	70-100	IV-III	NA	NA	NA	NA	NA	NA	NA	NA	NA
4	Glavatskyi, Neuro-Oncology, 2014 (30)	R	26	NA	NA	NA	IV-III	GBM-AA	8 GTR (30), 9 STR (35), 9 B (35)	9 (35)	17 (65)	NA	NA	NA	NA	0 (0)
5	Greenspoon, Onco Targets Ther, 2014 (20)	P	31	53; 36-75	NA	80; 60-90	IV	GBM	NA	31 (100)	0 (0)	NA	12.1	5	NA	31 (100)
6	Hasan, Front Oncol, 2015 (31)	R	19	56; 29-79	13:6	80; 40-100	IV	GBM	7 GTR (37), 8 STR (42), 4 NA (21)	19 (100)	19 (100)	16; 2-122	20.9	5	25	15 (79)
7	Lévy, Cancer/Radiothérapie, 2017 (32)	R	13	55	11:02	85; 65-100	IV-III	NA	6 GTR (46), 3 STR (23), 4 B (31)	13 (100)	13 (100)	11	23.6	NA	30	11 (85)
8	Pinzi, Neurol Sci, 2015 (33)	R	128	51; 18-79	80:48	60-100	IV-III	GBM-AA	38 GTR (30), 77 STR (60), 13 B (10)	128 (100)	84 (67)	15; 6-171	6.5	2	19	61 (48)
9	Torok, Technol Cancer Res Treat, 2011 (25)	R	14	58; 39-76	7:7	NA	IV	GBM	11 GTR (79), 3 PR (21)	14 (100)	14 (100)	8; 1-28	7	3	24	12 (86)
10	Villavicencio, Radiosurgery, 2010 (34)	R	26	55.5; 36-82	18:8	90; 50-100	IV	GBM	15 GTR (58), 9 STR (34), 2 B (8)	26 (100)	25 (96)	13; 5-89	7	2	NA	5 (19)
11	Yazici, J Neurooncol, 2014 (21)	R	37	37; 22-69	18:19	60-100	IV	GBM	23 GTR (62), 13 STR (35), 1 B (3)	37 (100)	37 (100)	15; 5-45	NA	5	30	11 (30)
12	Yoshikawa, Minim Invasive Neurosurg, 2006 (24)	P	25	54; 28-78	13:12	77.3; 30-90	IV-III	GBM-AA	3 GTR (12), 12 STR (48), 9 PR (36), 1 B (4)	14 (56)	14 (56)	NA	19.1	3	20.3	16 (64)

### Primary Outcomes

**Figure 2:**
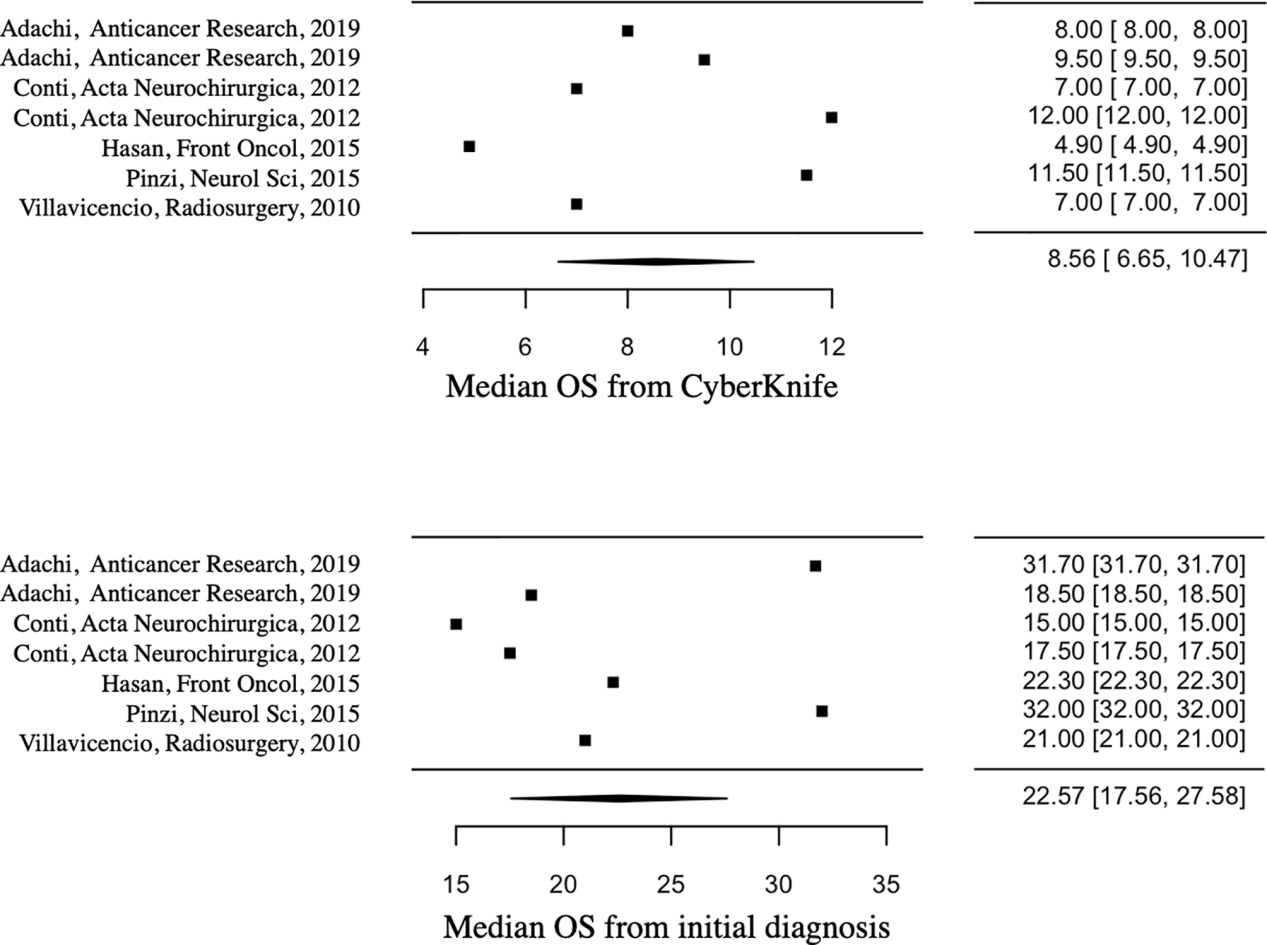
Forest plots showing median OS from initial diagnosis (below) and CK treatment (above).

Median OS from initial diagnosis and CK treatment was 22.57 months (95%CI=17.56-27.58) and 8.56 months (95%CI=6.65-10.47) respectively. **Figure 2** shows the median OS forest plots. Median TTP and median PFS from CK treatment were 6.68 months (95%CI=2.13-11.22) and 7.05 months (95%CI=1.30-12.79) respectively.

Concerning the WHO grade, the median OS from initial diagnosis was 19.88 months (95%CI=17-22.76) for WHO grade IV gliomas, compared to 48.35 months (95%CI=15.72-80.98) for WHO grade III gliomas. Median OS from CK treatment was 8.4 months (95%CI=6.35-10.45) for WHO grade IV gliomas, compared to 11 months (95%CI=5.12-16.88) for WHO grade III gliomas.

About the treatment, median OS from initial diagnosis was 25.4 months (95%CI=16.97-33.83) for patients who underwent CK plus CMT treatment, compared to 16.05 months (95%CI=13.99-18.11) for patients who underwent CK treatment alone; median OS from CK treatment was 9.52 months (95%CI=7.78-11.25) for patients who underwent combined treatment, compared to 4.44 months (95%CI=0-9.46) for patients who underwent CK treatment alone. Primary outcomes are reported in **Table 2**. We did not observe a positive correlation between median TTR and median OS from CK.

**Table 2:** Primary outcomes.

	Months (95%CI)
**Median Overall Survival**	
*From initial diagnosis*	22.57 (17.56-27.58)
* WHO grade IV*	19.88 (17.00-22.76)
* WHO grade III*	48.35 (15.72-80.98)
* CK alone*	16.05 (13.99-18.11)
* CK plus CMT*	25.40 (16.97-33.83)
*From CK treatment*	8.56 (6.65-10.47)
* WHO grade IV*	8.40 (6.35-10.45)
* WHO grade III*	11.00 (5.12-16.88)
* CK alone*	4.44 (0-9.46)
* CK plus CMT*	9.52 (7.78-11.25)
	
**Median Time To Progression**	
*From CK treatment*	6.68 (2.13-11.22)
	
**Median Progression Free Survival**	
*From CK treatment*	7.05 (1.30-12.79)

### Secondary Outcomes

**Figure 3:**
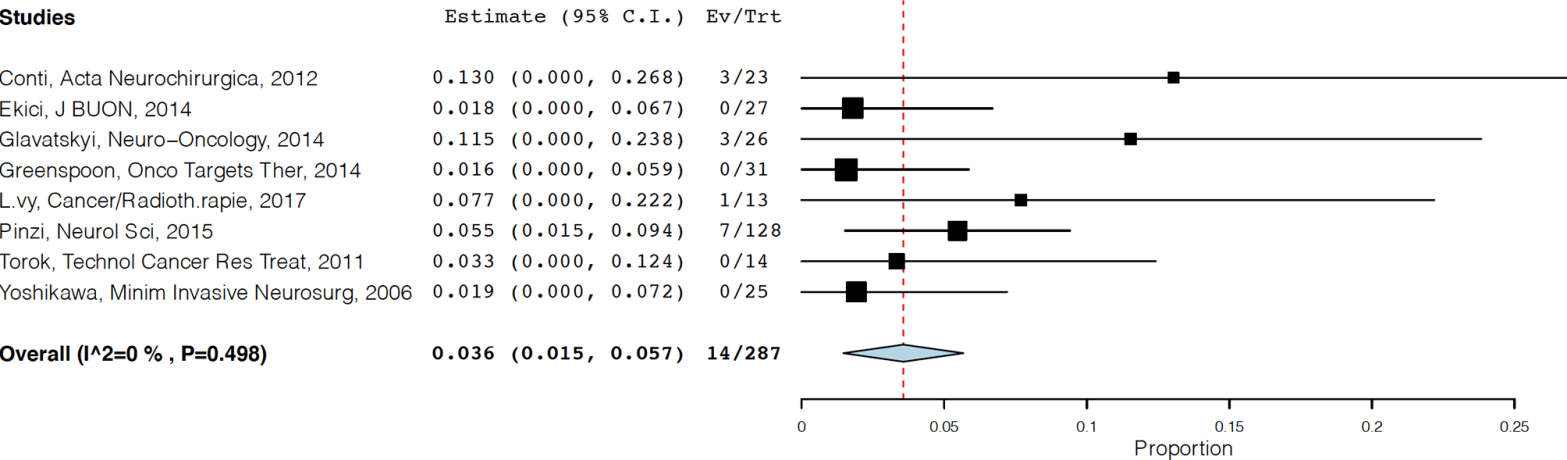
Forest plot showing rates of acute neurological side effects following CK treatment.

Rates of acute neurological and non-neurological side effects after CK treatment at recurrence were reported in 287 patients. The overall rate of the former was 3.6% (95%CI=1.5-5.7), while 13% for the latter (95%CI=0-26.1). Acute neurological effects included worsening of pre-existing symptoms, dizziness, nausea/vomiting, and neurological deterioration. Acute non-neurological effects included alopecia, fatigue, asthenia, and clinical deterioration. **Figure 3** shows the acute neurological side effects forest plot. Rates of corticosteroid dependency and radiation necrosis were reported in 271 patients and 306 patients respectively. The overall rate of corticosteroid dependency was 18.8% (95%CI=10.0-27.6), while the overall rate of radiation necrosis was 4.3% (95%CI=2.1-6.6). Rates of other toxicities were reported in 267 patients. The overall rate was 1.1% (95%CI=0.4-2.7) and these were hematological toxicities.

Rates of LR after CK treatment at recurrence were reported in 84 patients. The overall rate of PD was 37.9% (95%CI=26.5-49.3), followed by overall rates of 29.2% for NC (95%CI=15.7-42.7), 27.7% for PR (95%CI=18.2-37.2), and 2% for CR (95%CI=1.0-4.9).

Rates of NLs developed following CK treatment were reported in 61 patients, and the overall rate was 88.4% (95%CI=80.5-96.4). Rates of BR or RR were reported in 50 patients. The overall rate of BR was 75.9% (95%CI=64.3-87.6), compared to 17.7% for RR (95%CI=1.5-33.8). The secondary outcomes are summarized in **Table 3**.

**Table 3:** Secondary outcomes.

	Overall % (95%CI)
**Local Response**	
*Complete Response*	2.0 (1.0-4.9)
*Partial Response*	27.7 (18.2-37.2)
*No Change*	29.2 (15.7-42.7)
*Progressive Disease*	37.9 (26.5-49.3)
**Recurrence**	
*New Lesion*	88.4 (80.5-96.4)
*Beside Recurrence*	75.9 (64.3-87.6)
*Remote Recurrence*	17.7 (1.5-33.8)
**Complications**	
*Acute Neurological Side Effects*	3.6 (1.5-5.7)
*Acute non-Neurological Side Effects*	13.0 (0-26.1)
*Corticosteroid Dependency*	18.8 (10.0-27.6)
*Radiation Necrosis*	4.3 (2.1-6.6)
*Other Toxicities*	1.1 (0.4-2.7)

### Study Heterogeneity

I^2^ values were <50% indicating a lack of substantial heterogeneity for all the outcomes.

## Discussion

### Findings

The treatment strategy for patients harboring rMGs is still debated and no clear consensus has been achieved yet. Treatment modalities include surgery, CMT, RT, and combined treatments. Reirradiation with SRS can provide survival benefits with acceptable risks. Among diverse SRS machines currently available, we focused on the CK System. Our study’s primary aim was to establish the efficacy of CK treatment for rMGs, concerning survival and time to disease progression. Our secondary aims were to establish the local disease response, recurrence of disease, and toxicities. We performed a systematic review and meta-analysis of published studies on CK for rMGs and found several interesting findings.

#### Patients Characteristics

In our meta-analysis, we observed a male predominance (1.6:1). Recent evidence suggested that sex-associated biological features can play a role in MGs incidence, regardless of the age, race, and geographic location of patients (1, 35, 36). An average male-to-female ratio of 1.6:1 has been previously reported for MGs, with greater incidence in men (1, 37). The prevalence of MGs in males appeared to be related mainly to genetic dissimilarities and not only to the presence of sex hormones (38). Gender differences can be pivotal for developing tailored approaches to MGs and pursuing studies are taking into account sex differences for innovative treatment strategies (37).

#### Primary Outcomes

Median OS of rMGs without any treatment has been reported to range between 3 and 6 months (5). Reoperation of recurrent GBMs provides 3 to 5 months median survival, without a significant increase in morbidity and mortality, and is still limited to 10-30% of patients due to the infiltrative nature of the disease and the involvement of eloquent areas (39–42). Over the past years, reirradiation has been increasingly proposed as an alternative treatment strategy with successful results (43, 44). Among different reirradiation modalities, HFSRT and SRS have been reported to provide a median OS ranging from 8.6 to 18 months with acceptable side effects (45). Our meta-analysis on CK System revealed a median OS of 8.6 months (95%CI=6.65-10.47) from SRS treatment and 22.6 months (95%CI=17.56-27.58) considering survival from initial diagnosis. Median TTP and median PFS after CK treatment were comparable (6.7 vs 7.1 months), with a slightly longer median PFS as this outcome only differs for the inclusion of remote recurrence or death from any cause (23). Barbagallo et al. reported a similar mean PFS for patients with rMGs undergoing second surgery (7.7 months) (46). Randomized controlled trials (RCTs) are needed to provide more definitive answers about differences in particular treatment strategies for rMGs.

Regarding the grade of the disease, WHO grade IV rMGs showed a shorter median survival from CK treatment (8.4 months), compared to WHO grade III rMGs (11 months). Notably, Murai et al. reported a 3-year survival rate of 38% for re-irradiated patients with recurrent WHO grade III anaplastic ependymomas (AEs) and a median OS from CK treatment of 31.5 months (47). Therefore, treatment of recurrent AEs with CK System is a promising alternative, especially for deep-seated lesions or lesions located adjacent to eloquent areas (47–50).

The subgroup analysis of treatment strategy revealed a longer survival for patients undergoing CK plus CMT treatment (9.5 months) compare with patients undergoing CK treatment alone (4.4 months). Hu et al. previously reported that HFSRT combined with CMT confers a slight survival improvement for patients with rMGs compared with HFSRT alone (8.23-23.0 months vs 3.9-12.0 months) (51). In their meta-analysis including 388 patients, 3 out of the 7 selected studies presented statistically significant differences (P < 0.05) between these two treatment approaches, and 3 out of the 4 remaining studies showed a favorable survival for patients treated with combined therapy rather than HFSRT alone. Likewise, our meta-analysis suggests a longer survival for patients who undergo combined treatment, but we cannot ascertain the absence of confounding bias between the two groups and stratified RCTs would be needed for ultimate conclusions. Moreover, we were unable to perform qualitative subgroup analyses of the systemic agents used and the time of systemic therapy sessions with respect to CK treatment. Among the different agents used in the included studies, TMZ was the most reported CMT regimen (66 patients, 16%), followed by BEV in 22 patients (5%) and Interferon in 16 patients (4%). Administration of CMT was concomitant and/or after CK treatment in 199 patients (98%) and before CK treatment in 4 patients (2%). The latter received BEV-based salvage therapy prior to CK treatment (31). The most commonly used systemic therapies for rMGs include TMZ, nitrosoureas, and BEV (52–54). The combination of lomustine with BEV has shown improved PFS but not OS, and a higher toxicity rate compared with lomustine alone (55). Bevacizumab alone or in combination with chemotherapy agents such as lomustine or irinotecan has demonstrated a median survival time from recurrence around 9 months and radiographic response rates of approximately 30 to 40 percent (55, 56). Few reports described the combination of bevacizumab with HFSRT for recurrent GBMs with safe and effective results (57–59). This treatment strategy is under study in an ongoing larger randomized trial (60). Among the studies included in our meta-analysis, Hasan et al. showed a better survival for patients with recurrent GBMs treated with BEV either before or after CK treatment (31). Palmer et al. reported a slightly higher survival for patients with recurrent GBMs treated with HFSRT before BEV rather than BEV before HFSRT (13.9 vs 13.3 months) but stressed the importance of a randomized multi-institutional trial for more definite conclusions (61).

We did not observe a positive correlation between median TTR and median OS from CK. Likewise, Greenspoon et al. did not find a statistical difference in OS or PFS when stratifying by TTR (<12 months or >12 months) (20). Conversely, Yazici et al. reported improved survival for patients with a TTR of more than 12 months (21).

#### Secondary Outcomes

Our meta-analysis shows that CK is a relatively safe and effective treatment modality for rMGs. Rates of complications were relatively low. Corticosteroid dependency had the highest rate among the complications (18.8%), followed by acute non-neurological side effects (13%, including fatigue, alopecia, and clinical deterioration), and by radiation necrosis (4.3%). Notably, the authors of the included studies included steroid use among side effects only when requiring daily doses of dexamethasone > 4 mg for more than 8 weeks. However, we must acknowledge that current guidelines mention steroid use as a side effect from basic prescription (62). Larger re-irradiated tumors (maximum diameter greater than 4 cm) are more inclined to develop radiation necrosis (33, 63). Indeed, a crucial factor in developing radiation necrosis is the volume of the irradiated normal brain, which is relative to the tumor volume (64, 65). Radiation necrosis is known to occur in the normal brain when the normalized total dose (NTD) is greater than 100 Gy (66). Other authors reported that using a fractionated scheme aimed to maintain a normalized total dose (NTD)<100 Gy can reduce the risk of radionecrosis in larger tumors (26, 33). Conversely, rates of acute neurological effects (3.6%) such as worsening of pre-existing symptoms, dizziness, nausea/vomiting, neurological deterioration, and rates of hematological toxicities (1.1%) were the lowest. Acute side effects were higher in patients treated with large single fraction volumes, supporting the hypothesis that fractioned schemes may be safer for tumors larger than 4 cm in maximum diameter or proximal to eloquent areas (33). Hematological toxicities such as leukopenia and thrombocytopenia were mainly reported for patients who underwent CK treatment plus CMT (26). Although we meta-analyzed the side effects reported by the authors, it was not possible to grade toxicity because of a lack of uniformity among studies. Future trials should report the side effects according to standardized grading systems to enhance uniformity and facilitate interpretation of results (62, 67).

The analysis of LR at 4 weeks after CK treatment showed disease progression in 37.9% of cases, stability in 29.2%, reduction in 27.7%, and complete disappearance in 2%. Yoshikawa et al. reported a higher control rate (i.e. CR, PR, NC) for GBM patients than AA patients (63.6% vs 45.5%) (24). However, LR after CK was reported in a small overall cohort (84 patients), and this outcome should be validated by more extensive analyses. Moreover, true progression may often be indistinguishable from pseudoprogression (21). Pseudoprogression is a subacute effect of radiotherapy observed in the first 12 weeks after treatment, first described by Hoffman et al (68). It was pathologically defined by Chamberlain et al. as necrosis without evidence of tumor and appears as increased contrast enhancement following radiotherapy (69). These imaging findings are consequences of disruption of the blood-brain barrier and represent proof of radiation’s efficacy rather than progression or toxicity, indeed correlate with longer OS (21, 70, 71). Diagnosis of pseudoprogression is made during follow-up when stabilization or improvement of clinical and radiographic findings is observed (21). Instead, true progression within the first 12 weeks after radiotherapy, can only be defined if the majority of new enhancement is outside the radiation field or if there is pathological confirmation of PD (72).

The overall rate of NLs was considerably high (88.4%), with a greater rate of BR rather than RR (75.9% vs 17.7%). However, the rate of NLs was reported in only 61 patients, and the location of recurrence in only 50 patients overall. Therefore, this outcome needs to be corroborated by larger studies as well. Although it is known that recurrence of MGs appears mainly within 2 cm of the enhancing edge of the original tumor, Yoshikawa et al. reported the development of BR despite an initial high control rate (63.2% for GBM and 42.9% for AA controlled patients) (73). Despite surgery plays a key role in GBM recurrence, most of all for large volumes, CK radiosurgery has shown good results with a low rate of toxicity. Some aspects though remain unclear, such as radiation dose and fractionation.

A focus on the quality of life (QoL) is imperative given the poor prognosis and short life expectancy of patients with a diagnosis of rMGs. The QoL of MG patients is most often affected by the development of CMT/RT side effects, changes in physical functioning, and global health status (74). Unlike surgery and other SRS techniques, the CK treatment can be delivered without sedation and as an outpatient, which would help maximize the QoL. The primary and secondary end-points of our meta-analysis were based on outcomes reported by authors of the included studies. Therefore, we were unable to meta-analyze the effect of CK treatment on KPS, cognitive function, and QoL. However, Greenspoon et al. reported on the benefit of BEV in preventing toxicity and improving QoL of patients undergoing CK plus TMZ (20). Quality of Life after HFSRT for rMGs patients has been previously reported to remain stable for a median follow-up of 9 months (75). A subsequent study on high-dose reirradiation in selected patients with recurrent/progressive MGs found a stable QoL and improvement of activities of daily living (ADL) over a 1-year time period (76). Future studies should include KPS and QoL among their primary outcomes to evaluate the impact of CK treatment in life-limiting diseases such as rMGs.

### Limitations

Despite the significant number of patients included in our study, this meta-analysis was based primarily on a few single-center case series and thus has limitations inherent to single-center retrospective studies. Based on the data abstracted from the articles and provided by the authors of the included studies, we could not ascertain the number of patients undergoing repeat surgery and the EOR at recurrence. The different ways in which each study provided the confidence intervals and/or standard deviations did not allow the use of the standard errors for some of the outcomes in the meta-analysis. In such cases, a standard error of 0 was adopted for each study. This led to an imperfect approximation of the meta-analyzed outcome and its confidence interval. While we were able to perform subgroup analyses based on WHO grade and CMT at recurrence as well as analyzing the relationship between TTR and OS, we were unable to perform more granular analyses stratifying outcomes by other relevant variables such as the histotype, the PTV, irradiation dose, the number of fractions, the patients’ age and KPS. Moreover, we were unable to perform qualitative subgroup analyses of the CMT agents used and the time of CMT sessions with respect to CK treatment at recurrence. The assessment of LR was reported in a small cohort and differential diagnosis of lesions developed post-CK treatment can be misleading. Therefore, this outcome should be validated by more extensive analyses and future studies should focus on discrimination of lesions developed following CK treatment.

Nonetheless, to the best of our knowledge, this is the first meta-analysis providing helpful conclusions on the treatment of rMGs with the CK System and a potential start point for future studies.

## Conclusions 

Reirradiation of rMGs with the CK System has reasonable efficacy and provides encouraging survival rates. There is a better survival trend for WHO grade III lesions and for patients who undergo combined treatment with CK plus CMT. Treatment of rMGs with CK is a safe alternative, considering the low rates of complications. Larger and well-designed prospective studies are warranted to provide more accurate results.

## Data Availability Statement

The original contributions presented in the study are included in the article/supplementary material. Further inquiries can be directed to the corresponding author.

## Author Contributions

LDM: Writing - Original Draft, Term, Methodology, Validation, Resources, Project administration, Formal analysis, Investigation, Data Curation. LTB: Investigation, Resources, Data Curation, Formal analysis. AC: Resources, Writing - Review & Editing. KH: Resources, Writing - Review and Editing. VP: Resources, Writing - Review and Editing. TM: Resources, Writing - Review and Editing. RL: Resources, Writing - Review and Editing. SB: Resources, Writing - Review and Editing. MBM: Writing - Review and Editing. SMM: Writing - Review and Editing. MMF: Conceptualization, Validation, Writing - Review and Editing, Supervision, Project administration. All authors contributed to the article and approved the submitted version

## Conflict of Interest

The authors declare that the research was conducted in the absence of any commercial or financial relationships that could be construed as a potential conflict of interest.
